# Metal‐Free Synthesis of 1,3‐Divinylimidazolidin‐2‐One

**DOI:** 10.1002/open.202500618

**Published:** 2026-03-06

**Authors:** Sabine Lorenzen, Roland Graf, Nikolai V. Ignat’ev, Michael Schulte, Axel Delp, Fabio Calo, Maik Finze

**Affiliations:** ^1^ Institute of Inorganic Chemistry Institute for Sustainable Chemistry & Catalysis with Boron (ICB) University of Würzburg Würzburg Germany; ^2^ Merck Life Science KGaA Darmstadt Germany

**Keywords:** amines, structure elucidation, vinyl compounds, vinyl nitrogen compounds dehydrochlorination

## Abstract

A new one‐pot synthesis of 1,3‐divinylimidazolidin‐2‐one (**DVI**) starting from cheap and commercially available substances, i.e., di(chloroethyl)amine and phosgene or triphosgene was developed. The two‐step procedure provides a convenient access to **DVI** in high purity and good yield without the application of any catalytic system. Additionally, an alternative route that relies on the utilization of carbon dioxide for the preparation of **DVI** was assessed.

## Graphical abstract

The versatile building block 1,3‐divinylimidazolidin‐2‐one (DVI) was synthesized by a new route without the use of metal catalysts or gaseous acetylene, which was required according to previous methods. The synthesis utilizes commercially available inexpensive chemicals and enables the recycling of side products, allowing an economic and sustainable process.

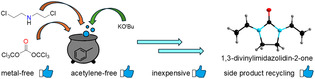

## 1 Introduction

1,3‐Divinylimidazolidin‐2‐one (**DVI**) is a well‐known cross‐linker in polymer chemistry [1, 2, 3, 4, 5, 6, 7]. Reports on the homo‐ and copolymerization of **DVI** date back to the early 70th of the last century [8, 9, 10]. Crawshaw and Jones [8] investigated the radical homopolymerization of **DVI** in *N*, *N*‐dimethylformamide initiated by azodi‐*iso*‐butyronitrile (AIBN). The polymer was separated by dropwise addition of the polymerization mixture to vigorously stirred diethyl ether. The polymer was obtained in almost quantitative yield as a colorless solid that was found not melt up to 335°C and to be practically insoluble in common organic solvents. The authors assumed cross‐linked structure for the obtained polymeric material. Later on, Corfield et al. [9] investigated the homopolymerization of **DVI** using different initiators, and found that di‐*tert*‐butyl peroxide provided a 90% conversion of **DVI** into a polymeric material after heating the reaction mixture to 130°C for 3 h in the absence of any solvent. Attempts to polymerize **DVI** using acid‐catalyzed initiation remained unsuccessful. Corfield et al*.* [10] studied the copolymerization of **DVI** with ethyl acrylate. The reactivity of **DVI** in the copolymerization with ethyl acrylate was low compared to 1‐ethyl‐3‐vinylimidazolidin‐2‐one.

The synthesis of the monomer **DVI** (*N*, *N*′‐divinylethyleneurea) was described for the first time in a US patent in 1951 [11]. The reaction of imidazolidin‐2‐one with acetylene in a closed vessel in the presence of elemental potassium resulted in a mixture of *N*, *N*′‐divinylethylene urea (**DVI**) and *N*‐monovinylethylene urea. This approach was based on Reppe‐type chemistry, which was developed at the end of the 1930s utilizing acetylene for a number of vinylation reactions [12]. The synthesis not only required acetylene under pressure but also strongly basic reaction conditions rendering this process difficult to handle. Safety precaution and equipment is needed to perform vinylation reactions under Reppe conditions. Over the last years, several reports were published on the application of calcium carbide as solid source for the in situ generation of acetylene [13, 14, 15, 16]. However, the use of this calcium carbide‐based technology for the vinylation of heterocyclic NH‐bases was reported not to be generally ideal for all substrates [17]. Later on, the synthesis of **DVI** using Ru‐based catalysts for the vinylation of imidazolidin‐2‐one with acetylene was optimized [18]. However, the reported yield of **DVI** was very low (15%).

Recently, Semina et al. [17] described the Ruthenium‐catalyzed preparation of **DVI** by vinylation of imidazolidin‐2‐one at low acetylene pressure. Again, the reported yield of **DVI** was very low (15%). It was increased to 40% by operating at an acetylene pressure of 1.5 bar and by the application of organocatalysts, i.e., commercially available phosphines such as tri‐*n*‐butylphosphine, at 140°C [19]. Nevertheless, a yield of 40% was too low to consider this reaction as an alternative method for the commercial production of **DVI**. Furthermore, this process still required handling of acetylene at high pressures as noted above.

**SCHEME 1:**
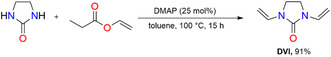
Synthesis of 1,3‐divinylimidazolidin‐2‐one (**DVI)** patented by BASF [20].

In 1996, BASF (Ludwigshafen, Germany) patented the preparation of **DVI** in 91% yield by the reaction of imidazolidin‐2‐one with vinyl propionate in toluene in the presence of 4‐*N*, *N*‐dimethylaminopyridine (DMAP) at 100°C (Scheme 1) [20]. However, this technology can not be considered acetylene free as the precursor vinyl propionate is obtained from acetylene and propionic acid in the presence of a ruthenium catalyst (5% wt. on carbon) [21].

**SCHEME 2:**
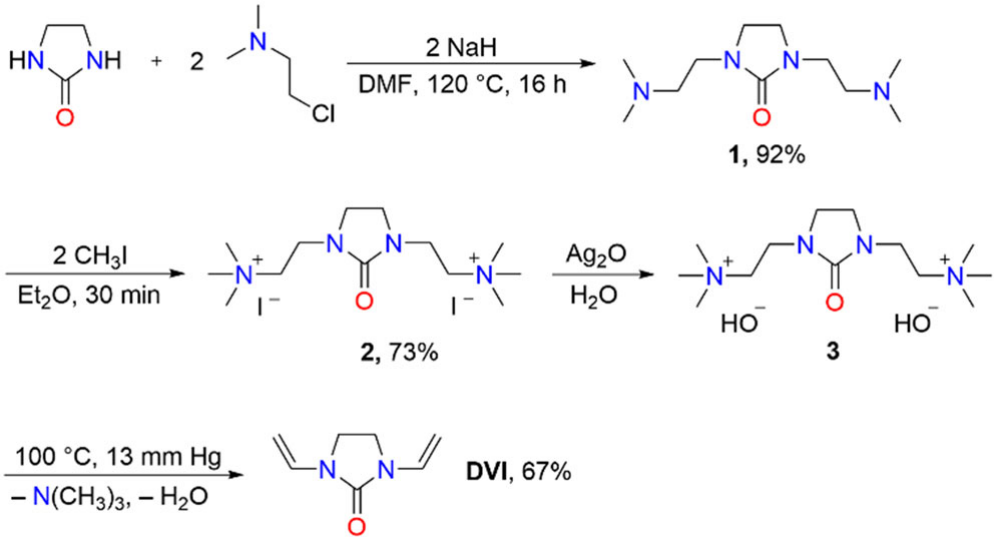
Synthesis of **DVI** according to a method described by Crawshaw et al*.* in 1971 [8].

Due to the requirements for the handling of acetylene as reagent in chemical reactions, an acetylene free synthesis of the valuable cross‐linking reagent for polymer chemistry **DVI** is highly desirable. In 1971, Crawshaw et al. [8] developed a four‐step synthesis of **DVI** that did not rely on acetylene (Scheme 2). In the first step, commercially available imidazolidin‐2‐one was alkylated with *β*‐dimethylaminoethyl chloride in the presence of sodium hydride in DMF to give 1,3‐di(*β*‐dimethylaminoethyl)imidazolidin‐2‐one (**1**). In the next step, compound **1** was methylated with methyl iodide to the diammonium salt **2**. The reaction of Ag_2_O with **2** gave the hydroxide salt **3**, which was directly thermolyzed to give **DVI** in a combined yield of 45% (Scheme 2).

**SCHEME 3:**
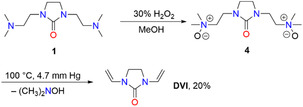
Synthesis of **DVI** by oxidation of 1,3‐di(*β*‐dimethylaminoethyl)imidazolidin‐2‐one [8].

In an alternative reaction sequence, 1,3‐di(*β*‐dimethylaminoethyl)imidazolidin‐2‐one (**1**) was oxidized with hydrogen peroxide to result in the corresponding *N*‐oxide **4**, which was converted into **DVI** at 100°C and reduced pressure under release of dimethyl hydroxyl amine (Scheme 3). However, the yield of **DVI** was low (20% over two steps).

Both protocols depicted in Schemes 2 and 3 are difficult to transfer into an industrial production of **DVI** due to (i) the multistep procedure, (ii) the use of expensive chemicals, and (iii) the relatively low yield of **DVI**. Therefore, the development of a simple method for the production of **DVI** without the necessity of heavy metal catalysts and/or acetylene as one of the starting compounds is of general interest, especially with respect to the production of **DVI**‐based materials for biotechnology applications that are used in pharma industry.

**SCHEME 4:**
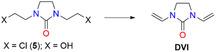
General approach for the synthesis of **DVI** without metal catalysts and/or acetylene developed in this study.

The aim of the present study was the development of a convenient synthesis for **DVI** from cheap and commercially available starting materials via well‐established, industrially scalable processes without the use of heavy metal catalysts and/or acetylene. In contrast to known methods for the preparation of **DVI**, which are based on vinylation of imidazolidin‐2‐one, we have developed the synthesis of **DVI** by formation of the vinyl groups in 1‐ and 3‐position of imidazolidin‐2‐one as the last step of the process using standard protocols for introduction of double bonds, namely by dehydration or dehydrohalogenation of the corresponding substrates (Scheme 4).

## 2 Results and Discussion

**SCHEME 5:**
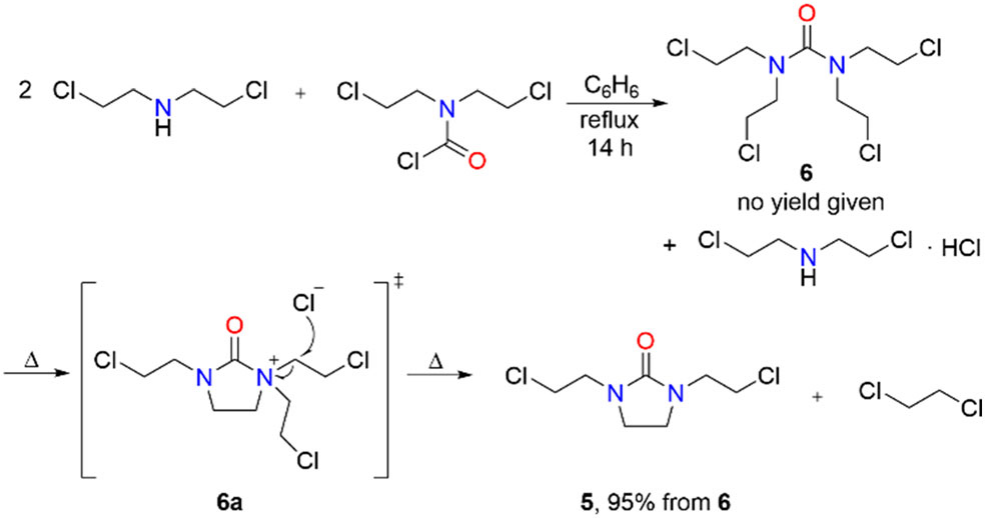
Synthesis of 1,3‐di(2‐chloroethyl)imidazolidin‐2‐one (**5**) in analogy to a method described by Settepani et al*.* [22].

The starting material for the dehydrochlorination reaction, 1,3‐di(2‐chloroethyl)imidazolidin‐2‐one (**5**), was prepared in 95% yield in two steps similar to a literature procedure (Scheme 5) [22]. *N*, *N*‐Di(2‐chloroethyl)carbamoyl chloride, which was synthesized from di(2‐chloroethyl)amine and carbonyl dichloride (phosgene) according to a protocol described by Childs et al. [23], was reacted with di(2‐chloroethyl)amine in dry benzene under reflux for 14 h (Scheme 5). Solid di(2‐chloroethyl)amine hydrochloride was removed by filtration (isolated in 99% yield) and the intermediate 1,1,3,3‐tetrakis(2‐chloroethyl)urea (**6**) was obtained by evaporation of benzene. Intermediate **6** was converted into 1,3‐di(2‐chloroethyl)imidazolidin‐2‐one (**5**) by vacuum distillation [22]. The quaternary amide **6a** was postulated as intermediate during the cyclization of 1,1,3,3‐tetrakis(2‐chloroethyl)urea (**6**) to give **5** [22]. Nucleophilic attack of a chloride ion led to carbon—nitrogen bond cleavage that resulted in release of 1,2‐dichloroethane, which was collected in a dry ice trap during the distillation and was isolated in 82% yield [22]. The byproduct di(2‐chloroethyl)amine hydrochloride can in principal be back‐converted into di(2‐chloroethyl)amine and recycled.

**SCHEME 6:**
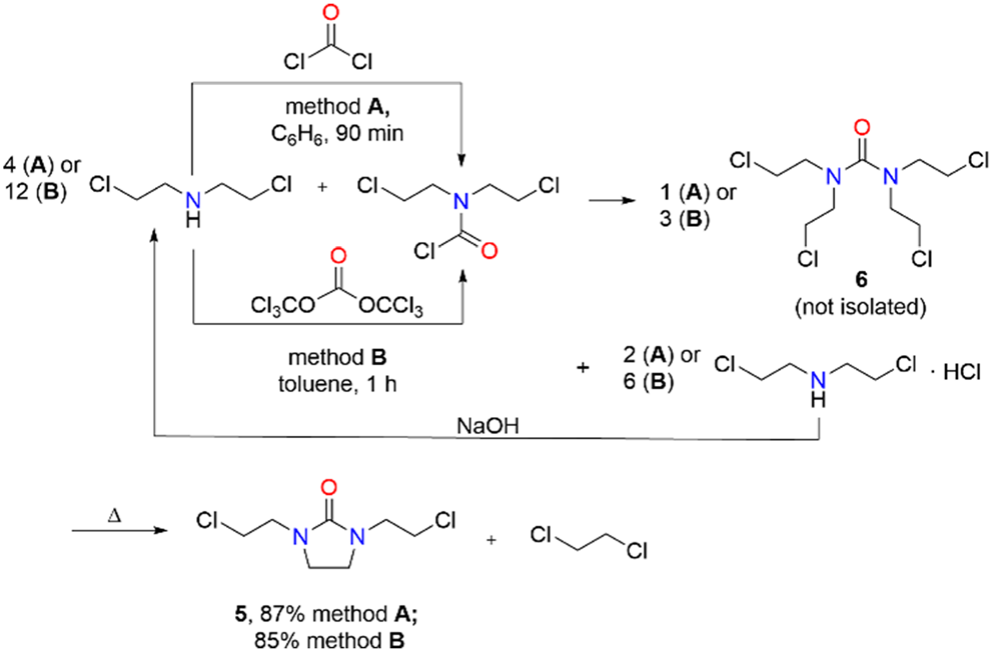
Synthesis of 1,3‐di(2‐chloroethyl)imidazolidin‐2‐one (**5**) by our optimized routes via phosgene (**Method A**) and triphosgene (**Method B**).

We have improved the synthesis of 1,3‐di(2‐chloroethyl)imidazolidin‐2‐one (**5**) by (i) combination of the single reaction steps into a one‐pot synthesis, (ii) use of di(2‐chloroethyl)amine as sole starting compound, and(iii) the alternative replacement of toxic gaseous phosgene by easy‐to‐handle solid triphosgene Cl_3_CO–C(O)–OCCl_3_, resulting in an overall much safer preparation of **5** (Scheme 6, method **B**). The application of toluene instead of benzene, which is undesirable or not allowed at all, is a further improvement of this procedure. Similarly to the protocol presented in Scheme 5, the byproduct di(2‐chloroethyl)amine hydrochloride can be back‐converted into the starting material di(2‐chloroethyl)amine and reused, leading to an improved atom efficiency.

**FIGURE 1:**
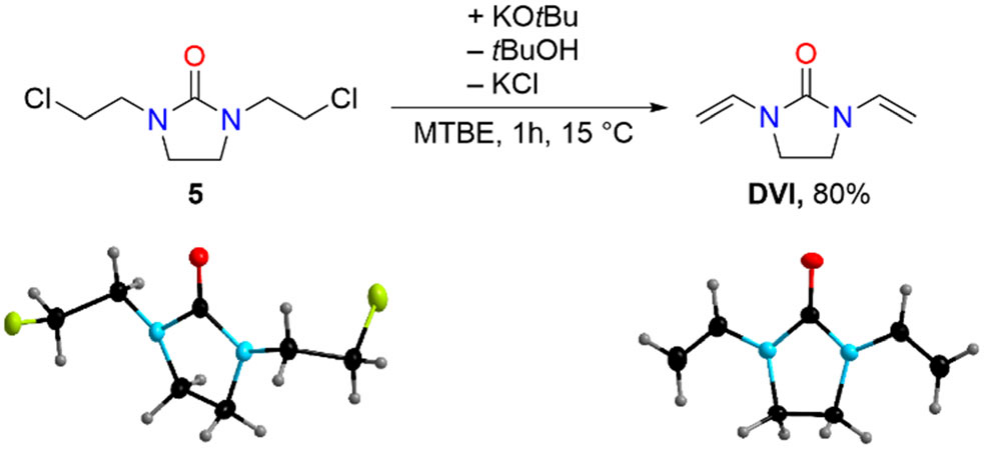
Synthesis of **DVI** from **5** and crystal structures of 1,3‐di(2‐chloroethyl)imidazolidin‐2‐one (**5**, left) and **DVI** (right; thermal ellipsoids set at 40% probability) determined by single‐crystal X‐ray diffraction (SC‐XRD). Selected bond lengths [pm] (mean value where applicable): **5**: C=O 123.0(2), N–C(O) 137.4(2), N–CH_2,(ring)_ 146.1(2), CH_2_–CH_2,(ring)_ 152.5(3), N–CH_2_ 144.7(2), CH_2_–CH_2_Cl 151.6(2), CH_2_–Cl 179.7(2); DVI: C=O 122.22(11), N–C(O) 137.05(12), N–CH_2,(ring)_ 145.51(12), CH_2_–CH_2,(ring)_ 153.69(13), N–CH 138.39(12), CH=CH_2_ 132.99(12).

Dehydrochlorination of 1,3‐di(2‐chloroethyl)imidazolidin‐2‐one (**5**) was carried out under basic conditions to avoid decomposition, oligomerization, or polymerization of the target compound **DVI**. The best yield and quality of **DVI** was achieved by performing the conversion of **5** into **DVI** with potassium‐*tert*‐butoxide in methyl *tert*‐butyl ether (MTBE) as solvent at 15°C. **DVI** was isolated in 80% yield as a colorless crystalline solid. Both compounds, **DVI** and its precursor 1,3‐di(2‐chloroethyl)imidazolidin‐2‐one (**5**) were characterized by single‐crystal X‐ray diffraction (SC‐XRD; Figure 1).

Importantly, both reaction steps shown in Scheme 6 and Figure 1 can be carried out as a one‐pot synthesis and **DVI** was obtained in a combined yield of 66%, which is practically the same yield as the one derived via a two‐step approach.

**SCHEME 7:**
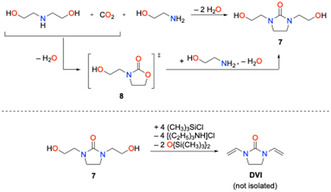
Synthesis of 1,3‐di(2‐hydroxyethyl)‐imidazolidin‐2‐one (**7**; top) [22] and dehydration of **7** to result in **DVI**.

In addition, an alternative entry toward **DVI** was evaluated that also relies on 1,3‐di(2‐hydroxyethyl)imidazolidin‐2‐one as starting compound (**7**; Scheme 7). Compound **7** was prepared according to a protocol described in the patent literature [24]. A mechanism was proposed for this three‐component reaction that includes 3‐(2‐hydroxyethyl)oxazolidone‐2 (**8**), which reacts with ethanol amine to the final product **7** (Scheme 7) [24]. The reaction, in principle requires very cheap chemicals only, i.e., diethanolamine, ethanol amine, and carbon dioxide. This green protocol utilizes CO_2_ and the sole byproduct is water. Compound **7** can be transformed into 1,3‐di(2‐chloroethyl)imidazolidin‐2‐one (**5**) using thionyl chloride [22] providing an alternative convenient entry to the precursor for the dehydrochlorination‐route to **DVI** (Figure 1). In addition, dehydration of **7** was investigated under different acidic and basic conditions. Most of these attempts failed and no reaction was observed. When **7** was treated with the ionic liquid [EMIm]^+^(HSO_4_)^–^ ([EMIm]^+^ = 1‐ethy‐3‐methylimidazolium cation) formation of a polymeric material was observed. Presumably, dehydration occurred but the **DVI** formed immediately polymerized. Dehydration was achieved with a mixture of trimethylsilyl chloride and triethylamine (Scheme 7) according to ^1^H NMR spectra. However, further optimization is necessary since isolation of pure **DVI** was not possible, so far.

## 3 Conclusion

**DVI** was prepared starting from cheap and commercially available substances, i.e., di(chloroethyl)amine and triphosgene. This simple two‐step procedure provides convenient access to **DVI** in high purity and good yield without using acetylene and/or metal catalysts. In addition, a promising “green” protocol for the preparation of **DVI** utilizing CO_2_ was evaluated.

## 4 Experimental

### 4.1 General

^1^H, ^13^C, and ^15^N spectra were recorded at 298 K on a Bruker Avance 500 NMR spectrometer or a Bruker Avance Neo 400 spectrometer. NMR signals were referenced against (CH_3_)_4_Si (^1^H and ^13^C) with *δ*(^13^C) = 25.145020 MHz, MeNO_2_ with *δ*(^15^N) = 10.136767 MHz [25]. ^1^H and ^13^C chemical shifts were calibrated against the residual solvent signal and the solvent signal, respectively [26]. IR spectra were recorded on a Bruker Alpha‐FT‐IR spectrometer with an apodized resolution of 2 cm^–1^ in the attenuated total reflection (ATR) mode in the region of 4000–500 cm^–1^ using a setup with a diamond crystal. Raman spectra were recorded on a Bruker MultiRAM FT Raman spectrometer with an apodized resolution of 2 cm^–1^ using the 1064 nm excitation line of a Nd:YAG laser on samples contained in melting point capillaries in the region of 3500–400 cm^–1^. High resolution mass spectrometry (HRMS), electrospray ionization (ESI), and atmospheric pressure solids analysis probe (ASAP) spectra were recorded using an Exactive Plus mass spectrometer with an Orbitrap (Thermo Scientific) equipped with an ESI source (3.5 kV spray voltage) or an atmospheric pressure chemical ionization (APCI) probe (4.0 µA discharge current). Elemental analyses (C, H, N) were performed either with a Euro EA3000 instrument (HEKA‐Tech, Germany) or with an Elementar Vario MICRO cube instrument (Elementar Analysensysteme, Germany). Thermal analyses were performed with a DSC 204 F1 Phoenix (Netzsch) in the temperature range of 0°C–550°C with a heating rate of 10 K min^−1^. Karl‐Fischer titration was carried out at room temperature with an 831 KF Coulometer Metrohm and a double Pt wire electrode for coulometry using the reagent Aquastar CombiCoulomat fritless. GC–MS analyses were performed using a Thermo Fisher Scientific Trace 1310 gas chromatograph (column: TG‐SQC 5% phenyl methyl siloxane, 15 m, Ø 0.25 mm, film 0.25 μm; injector: 220°C; oven: 40°C (5 min), 40°C–280°C (20°C min^−1^); carrier gas: He (1.2 mL min^−1^)) equipped with a Thermo Fisher Scientific AI 1310 autosampler/injector and a Thermo Fisher Scientific ISQ QD single quadrupole detector operating in EI (electron ionization) mode.

### 4.2 Chemicals

All standard chemicals were obtained from commercial sources. Solvents were dried according to standard protocols and stored in flasks equipped with valves with PTFE stems (Rettberg, Göttingen) under an argon atmosphere. 1,3‐di(2‐hydroxyethyl)imidazolidin‐2‐one (**7**) was prepared according to a literature procedure [24].

### 4.3 Synthesis

#### 4.3.1 1,3‐Di(2‐chloroethyl)imidazolidin‐2‐one (5), Method A

Di(chloroethyl)amine hydrochloride (51.3 g, 287 mmol, 1.0 eq.) was suspended in benzene (350 mL) and a sodium hydroxide solution in water (1 mol L^–1^, 345 mL, 345 mmol, 1.2 eq.) was added. The mixture was stirred at room temperature for 90 min until full conversion to di(chloroethyl)amine as monitored by GC/MS. The two phases were separated, and the aqueous phase was extracted with benzene (2 ×15 mL). The combined organic phases were washed with water (1 × 40 mL) and dried with Na_2_SO_4_. All solids were filtered off and the solvent was in part removed under reduced pressure (45°C, 10 mbar). The remaining solution (259 g, 14.3% w/w di(chloroethyl)amine in benzene, 260 mmol, 91% yield) was analyzed by NMR spectroscopy and Karl Fischer titration (water content 34 ppm). ^1^H NMR (400 MHz, d_6_‐acetone, benzene): *δ* = 2.09 (t, 4H, *J*_H,H_ = 6.1 Hz, C*H*_2_), 1.40 (t, 4H, *J*_H,H_ = 6.1 Hz, C*H*_2_), N*H* not observed.

A solution of phosgene in toluene (31.5 g, 20% w/w, 63.7 mmol, 1.0 eq.) was slowly added to a freshly prepared solution of di(chloroethyl)amine in benzene (259 g, 14.3% w/w, 260 mmol, 4.08 eq.) resulting in the immediate formation of a colorless solid. The reaction mixture was heated to 110°C for 16 h. After cooling to room temperature, the precipitate, di(chloroethyl)amine hydrochloride, was filtered off and washed with benzene (2 × 20 mL). The organic phases were combined and all volatiles were removed under reduced pressure. The residue was heated to 150°C to result in a colorless solid (11.7 g, 55.4 mmol, 87% yield calculated for phosgene). ^1^H NMR (400 MHz, d_6_‐acetone): *δ* = 3.69 (t, 4H, *J*_H,H_ = 6.4 Hz, C*H*_2_), 3.48 (t, 4H, *J*_H,H_ = 6.4 Hz, C*H*_2_), 3.48 ppm (s, 4H, C*H*_2_C*H*_2_).

#### 4.3.2 1,3‐Di(2‐chloroethyl)imidazolidin‐2‐one (5), Method B

Di(chloroethyl)amine hydrochloride (180 g, 1.01 mol, 1.0 eq.) was suspended in toluene (800 mL). An aqueous sodium hydroxide solution (2 mol L^–1^, 550 mL, 1.10 mol, 1.1 eq.) was added and the mixture was stirred at room temperature for 1 h. Both phases were separated, and the aqueous phase was extracted with toluene (2 × 150 mL). The combined organic phases were washed with distilled water (5 × 100 mL) and dried with magnesium sulfate. After filtration part of the toluene was distilled off at reduced pressure (8 mbar) at 45°C. According to ^1^H NMR spectroscopy, the residue (1077 g) was composed of 12.0% w/w of di(chloroethyl)amine in toluene (908 mmol, 90% yield). Karl‐Fischer titration showed the presence of a small quantity of water (5 ppm) in this mixture, which had no influence on the subsequent reactions. ^1^H NMR (400 MHz, d_6_‐acetone, toluene): *δ* = 2.17 (t, 4H, *J*_H,H_ = 6.1 Hz, CH_2_), 1.50 (t, 4H, *J*_H,H_ = 6.1 Hz, C*H*_2_), N*H* not observed.

A solution of triphosgene (purity 98%, 22.5 g, 74.3 mmol, 1.0 eq.) in toluene (450 mL) was added to the freshly prepared solution of di(chloroethyl)amine in toluene (12.0% w/w in toluene, 908 mmol, 12.2 eq) and the reaction mixture was stirred under reflux for 16 h. After cooling to room temperature, the solid di(chloroethyl)amine hydrochloride that had formed was filtered off and washed with toluene (3 × 50 mL). The combined organic phases were washed with hydrochloric acid (0.1 mol L^–1^, 3 × 150 mL). During this process, additional di(chloroethyl)amine hydrochloride precipitated that was removed by filtration. The filtrate was washed with distilled water (5 × 100 mL) and dried with magnesium sulfate. After filtration, all volatiles were distilled off at reduced pressure (77 mbar) at 45°C. The crude product 1,3‐di(2‐chloroethyl)imidazolidin‐2‐one (**5**; 65.1 g) was further purified by short‐path distillation at 1 × 10^–1^ mbar and at elevated temperature. The first fraction that was collected at temperatures up to 100°C solely contained toluene. 1,3‐Di(2‐chloroethyl)imidazolidin‐2‐one (**5**) was collected in the temperature range of 135°C–140°C that solidified upon condensation to yield **5** as a colorless solid (39.8 g, 189 mmol, 85% yield). ^1^H NMR (400 MHz, d_6_‐acetone): *δ* = 3.69 (t, 4H, *J*_H,H_ = 6.9 Hz, C*H*_2_), 3.48 (t, 4H, *J*_H,H_ = 6.9 Hz, C*H*_2_), 3.48 ppm (s, 4H, C*H*_2_C*H*_2_). ^1^H NMR (400 MHz, CD_2_Cl_2_): *δ* = 3.63 (t, 4H, *J*_H,H_ = 6.2 Hz, C*H*_2_), 3.49 (t, 4H, *J*_H,H_ = 6.2 Hz, C*H*_2_), 3.45 ppm (s, 4H, C*H*_2_C*H*_2_). ^13^C{^1^H} NMR (126 MHz, d_6_‐acetone): *δ* = 161.2 (s, 1C, CO), 46.9 (s, 2C, CH_2_), 44.0 (s, 2C, CH_2_), 42.9 ppm (s, 2C, CH_2_). ^15^N NMR (^15^N‐^1^H HMBC, CD_2_Cl_2_): *δ* = –299.5 ppm (s, 2N, NC). Elemental analysis calcd (%) for C_7_H_12_N_2_Cl_2_O: C 39.83, H 5.73, N 13.27; found, C 39.45, H 5.86, N 13.02. HRMS (ASAP^+^) m/z, calcd for C_7_H_12_N_2_O_2_Cl_2_^+^: 211.0399 (100%), 213.0370 (64.0%); found: 211.0395 (100%), 213.0364 (64.0%).

#### 4.3.3 1,2‐Divinylimidazolidin‐2‐one (DVI)

1,3‐Di(2‐chloroethyl)imidazolidin‐2‐one (**5**) (39.8 g, 189 mmol, 1.0 eq.) was dissolved in MTBE (350 mL) at 35°C. This solution was cooled to 10°C and a suspension of potassium *tert*‐butoxide (46.5 g, 414 mmol, 2.2 eq.) in MTBE (450 mL) was added dropwise within 50 min under vigorous stirring, keeping the temperature of the reaction mixture at 10°C–15°C. After complete addition, the reaction mixture was stirred for another hour at 15°C until the GC/MS analysis showed full conversion of **5**. Subsequently, distilled water (200 mL) was added to the reaction mixture while stirring. The organic phase was separated and washed with cold bidistilled water (5 × 100 mL) until neutral pH‐value was reached. The organic solution was dried with magnesium sulfate, filtered, and the solvent was removed under reduced pressure. **DVI** was obtained as a colorless solid (21.0 g, 152 mmol, 80% yield). The total yield of **DVI** starting from di(chloroethyl)amine hydrochloride according to **Method B** is 68%. ^1^H NMR (400 MHz, CD_3_CN): *δ* = 6.93 (dd, 2H, *J*_H,H_ = 15.9, *J*_H,H_ = 9.0 Hz, C*H*), 4.25 (d, 2H, *J*_H,H_ = 9.0 Hz, C*H*), 4.24 (d, 2H, *J*_H,H_ = 15.9 Hz, C*H*), 3.59 ppm (s, 4H, C*H*_2_C*H*_2_). ^13^C{^1^H} NMR (126 MHz, CD_3_CN): *δ* = 154.0 (s, 1C, CO), 131.0 (s, 2C, C=C), 90.7 (s, 2C, C=C), 40.2 (s, 2C, NCH_2_) ppm. ^15^N NMR (^15^N‐^1^H HMBC, CD_3_CN): *δ* = −268.0 ppm (s, 1N, NCH_2_). Elemental analysis calcd (%) for C_7_H_10_N_2_O: C 60.85, H 7.30, N 20.28; found: C 60.67, H 7.41, N 20.04. HRMS (ASAP^+^) m/z, calcd for C_7_H_11_N_2_O^+^: 139.0866; found: 139.0862.

#### 4.3.4 One‐pot synthesis of 1,2‐Divinylimidazolidin‐2‐one (DVI)

A solution of phosgene in toluene (31.5 g, 20% w/w, 63.7 mmol, 1.0 eq.) was slowly added to a freshly prepared solution of di(chloroethyl)amine in benzene (259 g, 14.3% w/w, 260 mmol, 4.08 eq.) resulting in the immediate formation of a colorless solid. The reaction mixture was heated at 110°C for 16 h. After cooling to room temperature, the precipitated di(chloroethyl)amine hydrochloride was filtered off and washed with benzene (2 × 20 mL). All volatiles were removed under reduced pressure. The residue was heated to 150°C for 1 h to initiate the ring closure reaction to give 1,3‐di(2‐chloroethyl)imidazolidin‐2‐one (**5**; 9.70 g, 46.0 mmol, 72% yield) as confirmed by NMR spectroscopy. A fraction of 1,3‐di(2‐chloroethyl)imidazolidin‐2‐one (**5**; 3.16 g) was used for the subsequent conversion into **DVI** while the remainder of **5** (6.54 g) was kept and used for different other experiments.

Compound **5** (3.16 g, 15.0 mmol, 1.0 eq.) was dissolved in dioxane (5 mL) and a solution of potassium *tert*‐butoxide (3.37 g, 30.0 mmol, 2.0 eq.) in dioxane (30 mL) was added at room temperature dropwise within 10 min under vigorous stirring. The reaction mixture was stirred for another hour until the GC/MS analysis indicated full conversion of **5**. After centrifugation of the reaction mixture (15 min at 2000 rotations per min), the solution was separated and the solid was suspended in dioxane (20 mL) and centrifuged once more at 2000 rotations per min for 15 min. The liquid organic phases were combined, and the solvent was removed under reduced pressure. **DVI** was obtained as an off‐white solid (1.90 g, 13.8 mmol, 92% yield calculated for **5**). The total yield of **DVI** starting from phosgene is 66%. The NMR spectroscopic data of **DVI** obtained via the one‐pot procedure are identical to those of **DVI** obtained via methods **A** and **B**.

## Supporting Information

Additional supporting information can be found online in the Supporting Information section. The authors cited additional references within the Supporting Information [27, 28, 29, 30, 31]. **Supporting Fig. S1a:** ^1^H NMR spectrum of di(chloroethyl)amine in toluene with d_6_‐acetone as internal standard. **Supporting Fig. S1b:** ^1^H NMR spectrum of di(chloroethyl)amine in toluene with d_6_‐acetone as internal standard. **Supporting Fig. S2:** DSC curve of di(chloroethyl)amine. **Supporting Fig. S3a:** ^1^H NMR spectrum of **5** in d_6_‐acetone. **Supporting Fig. S3b:** ^1^H NMR spectrum of **5** in d_6_‐acetone. **Supporting Fig. S4a:** ^1^H NMR spectrum of **5** in dichloromethane. **Supporting Fig. S4b:** ^1^H NMR spectrum of **5** in dichloromethane. **Supporting Fig. S5:** ^13^C{^1^H} NMR spectrum of **5** in d_6_‐acetone. **Supporting Fig. S6:** IR (top) and Raman spectrum (bottom) of 5. **Supporting Fig. S7:** DSC curve of 5. **Supporting Fig. S8a:** ^1^H NMR spectrum of **DVI** in CD_3_CN. **Supporting Fig. S8b:** ^1^H NMR spectrum of **DVI** in CD_3_CN. **Supporting Fig. S9:** ^13^C{^1^H} NMR spectrum of **DVI** in CD_3_CN. **Supporting Fig. S10:** IR (top) and Raman spectrum (bottom) of **DVI**. **Supporting Table S1:** Selected crystal data and details of the refinement of the crystal structures of 1,3‐di(2‐chloroethyl)imidazolidin‐2‐one (**5**) and 1,3‐divinylimidazolidin‐2‐one (**DVI**).

## Funding

This work was supported the Merck Life Science KGaA.

## Conflicts of Interest

The authors declare no conflicts of interest.

## Supporting information

Supplementary Material

## Data Availability

The data that support the findings of this study are available in the supplementary material of this article.
