# Supplementary material for: Metal‐Free Synthesis of 1,3‐Divinylimidazolidin‐2‐One
Source: ChemistryOpen. 2026 Mar 6;15(3):e202500618. doi: 10.1002/open.202500618 (PMC12964074; doi:10.1002/open.202500618)
Supplement: Supplementary file 1 — Supplementary Material [file OPEN-15-e202500618-s001.pdf]

Supporting Information

Metal-free Synthesis of 1,3-Divinylimidazolidin-2-on (DVI)

Sabine Lorenzen,[a] Roland Graf,[a] Nikolai V. Ignat'ev,[a,c] Michael Schulte,[b] Axel Delp,[b] Fabio Calo,[b] and Maik Finze[a,*]

[a] Sabine Lorenzen, Roland Graf, Nikolai V. Ignat'ev, Maik Finze
University of Würzburg, Institute of Inorganic Chemistry, Institute for Sustainable Chemistry & Catalysis with Boron (ICB), Am Hubland, 97074 Würzburg (Germany)
E-mail: maik.finze@uni-wuerzburg.de
Homepage: https://go.uniwue.de/finze-group

[b] Michael Schulte, Axel Delp, Fabio Calo
Merck Life Science KGaA, Frankfurter Str. 250, 64293 Darmstadt (Germany)

[c] Nikolai V. Ignat'ev
Consultant, Merck Life Science KGaA, 64293 Darmstadt (Germany)

Experimental Spectra

Di(chloroethyl)amine

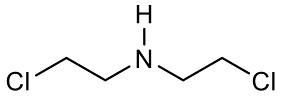

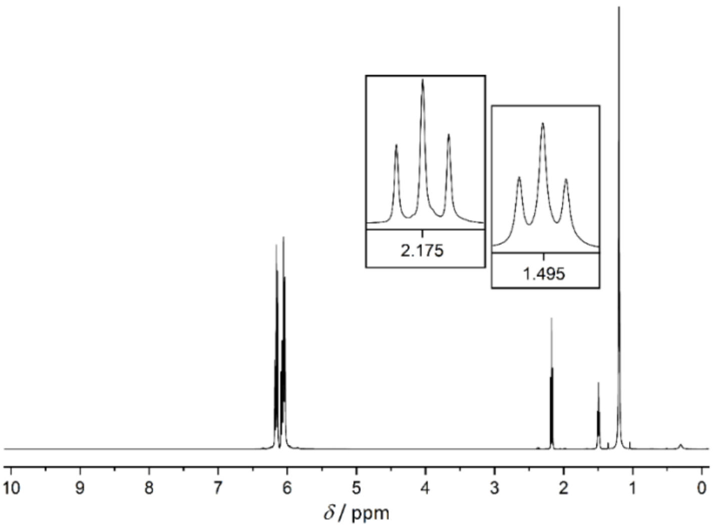

Fig. S1a. ^{1}H NMR spectrum of di(chloroethyl)amine in toluene with d_6-acetone as internal standard.

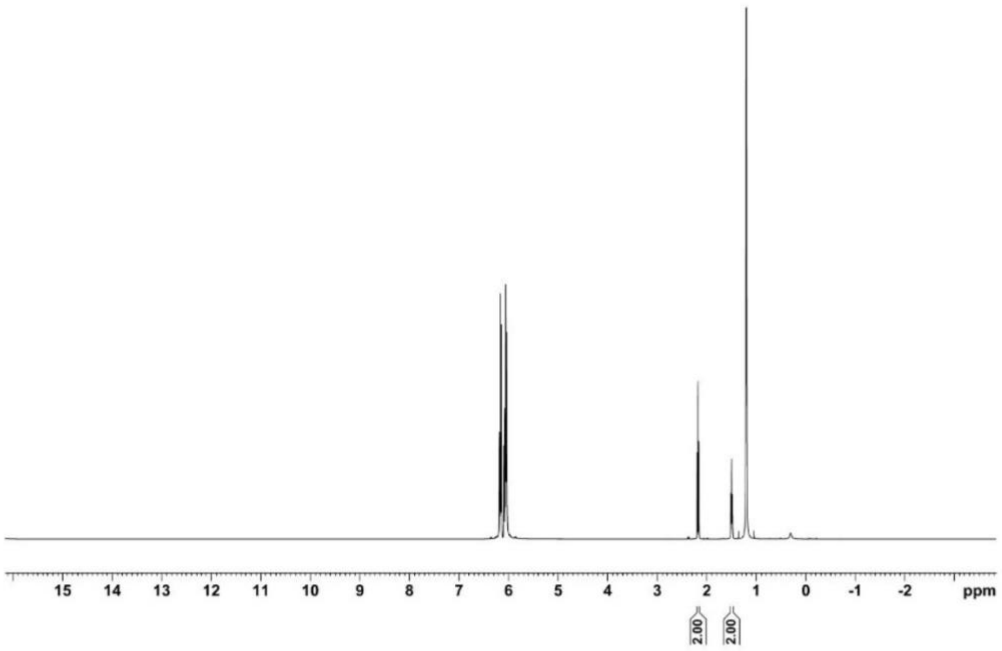

Fig. S1b. ^{1}H NMR spectrum of di(chloroethyl)amine in toluene with d_6-acetone as internal standard.

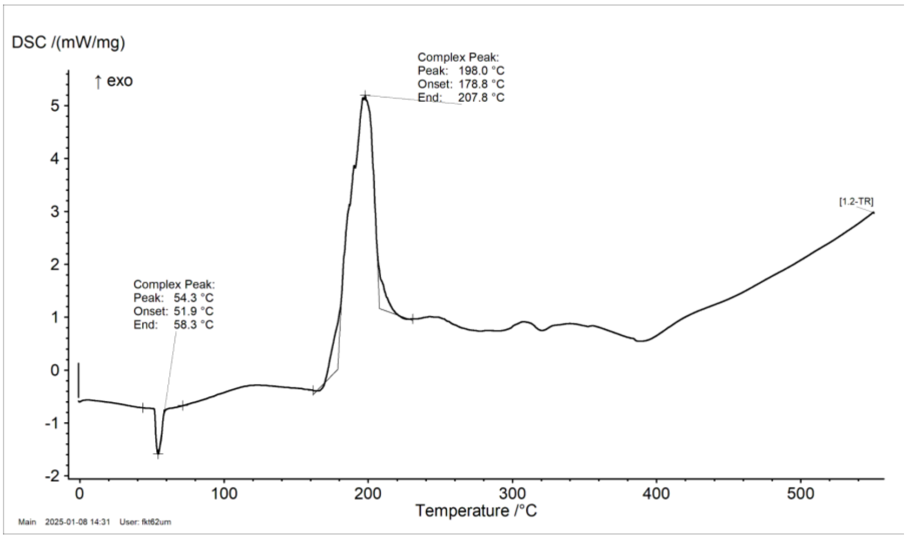

Fig. S2. DSC curve of di(chloroethyl)amine.

1,3-Di(2-chloroethyl)imidazolidin-2-one (5)

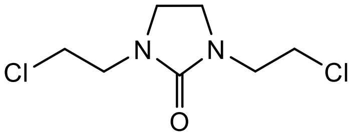

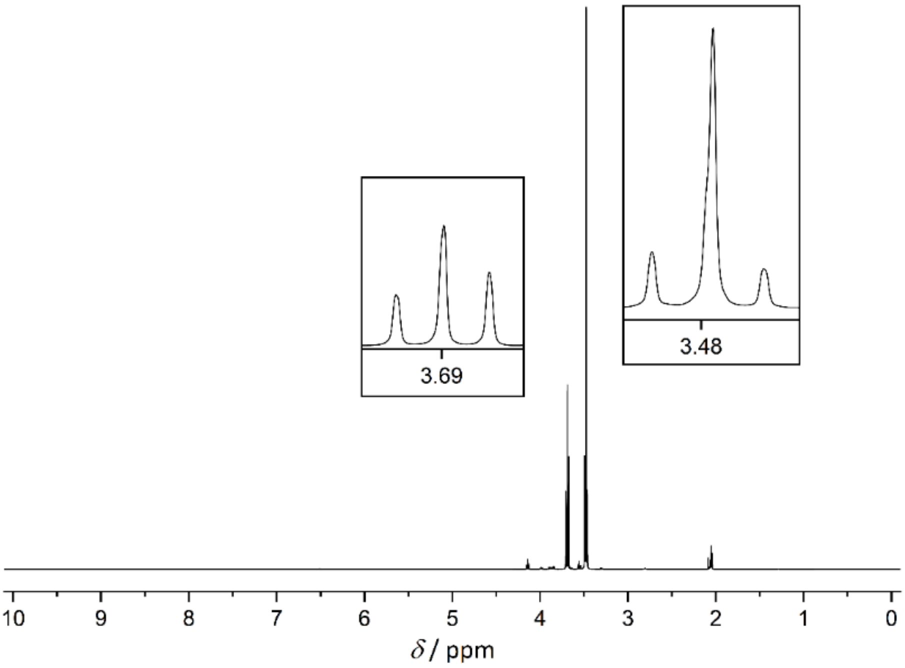

Fig. S3a. ^{1}H NMR spectrum of **5** in d_6-acetone.

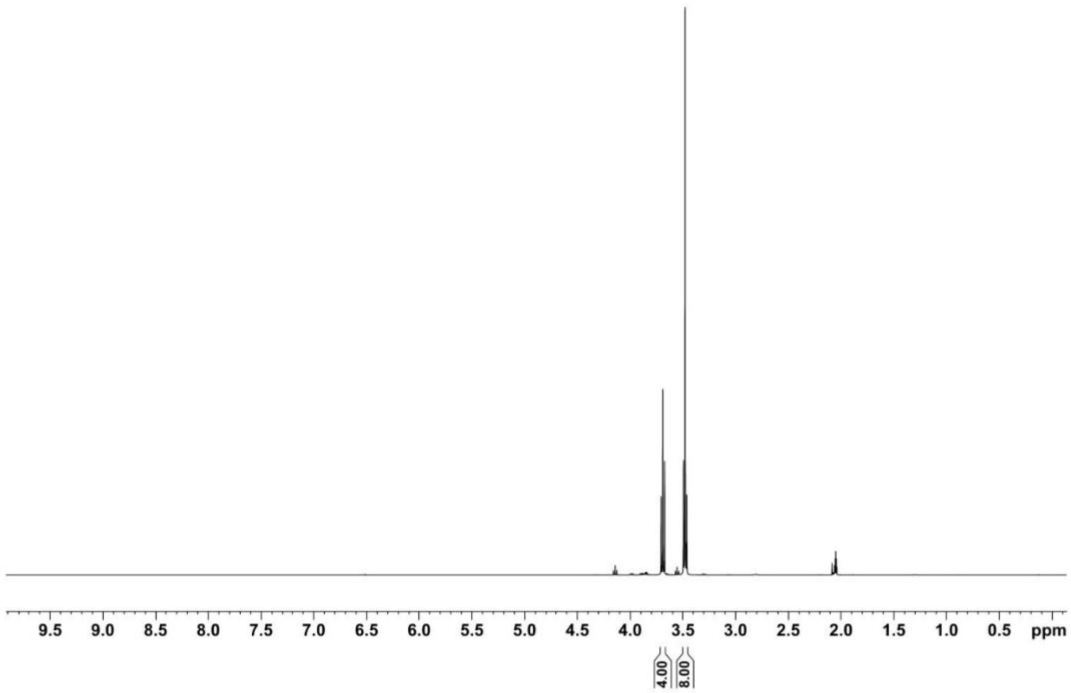

Fig. S3b. ^{1}H NMR spectrum of **5** in d_6-acetone.

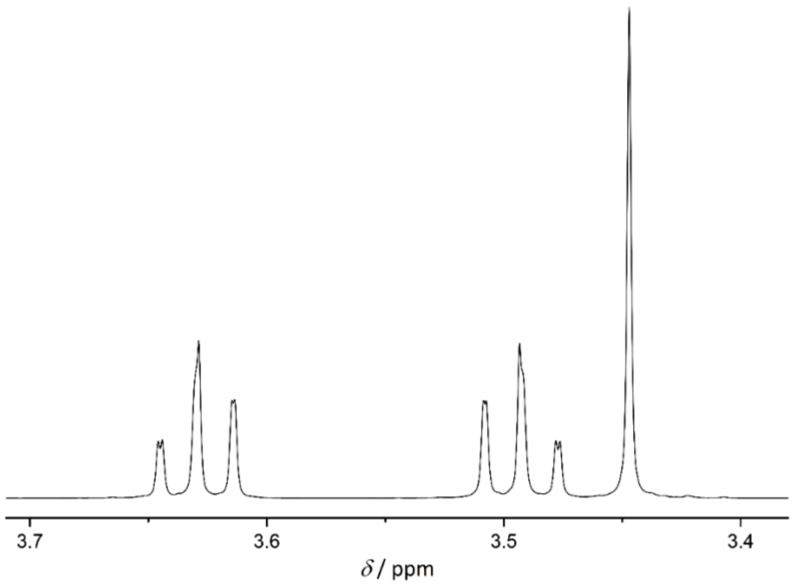

Fig. S4a. ^{1}H NMR spectrum of **5** in dichloromethane.

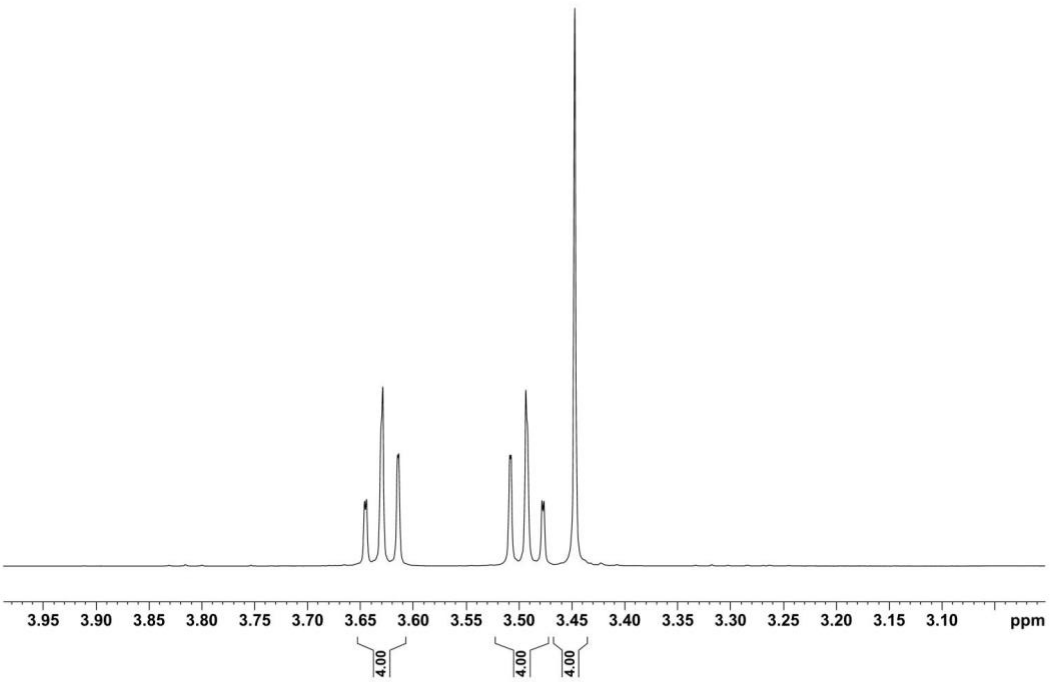

Fig. S4b. ^{1}H NMR spectrum of **5** in dichloromethane.

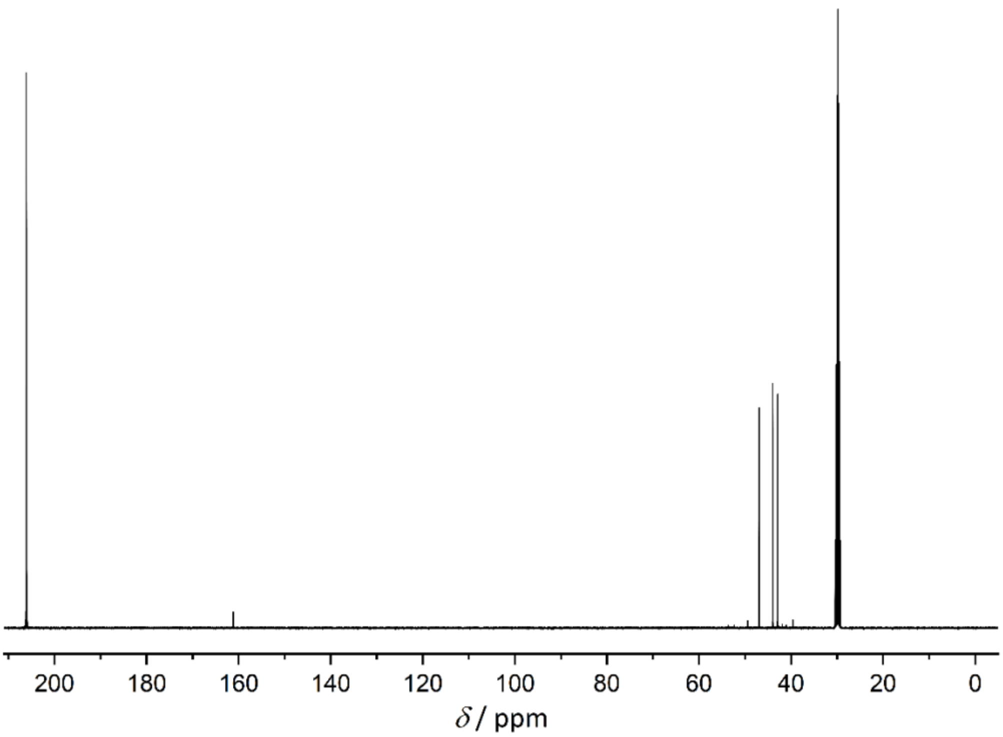

Fig. S5. $^{13}C\{^{1}H\}$ NMR spectrum of **5** in d_6-acetone.

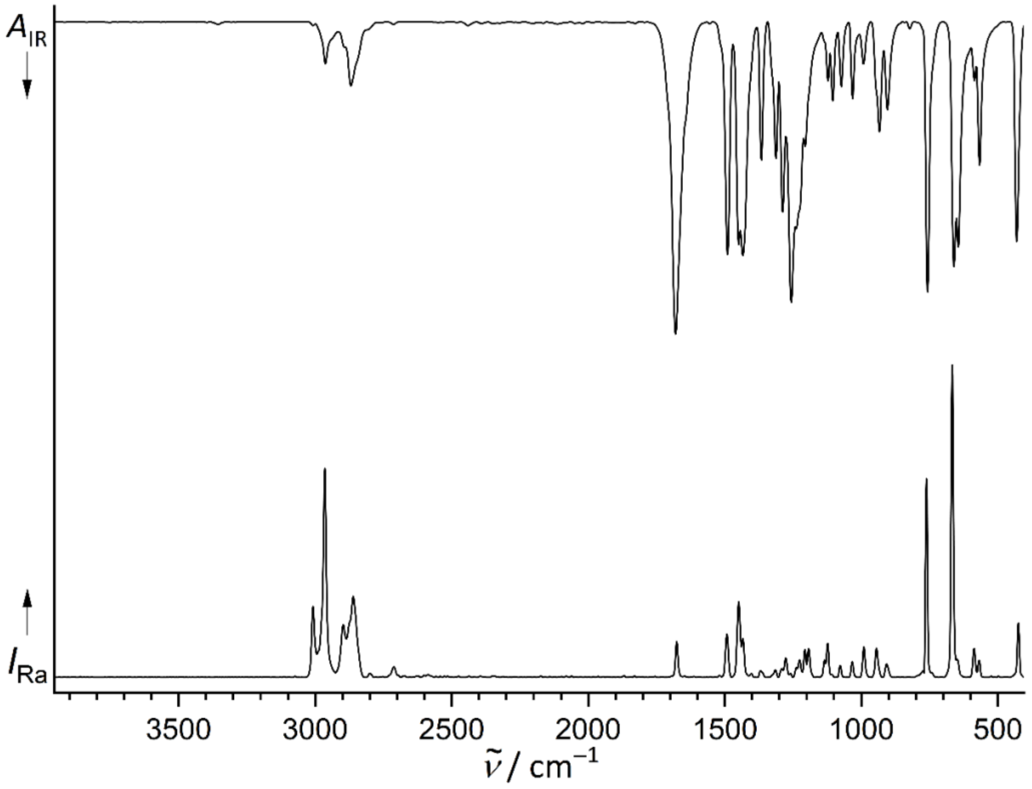

Fig. S6. IR (top) and Raman spectrum (bottom) of **5**.

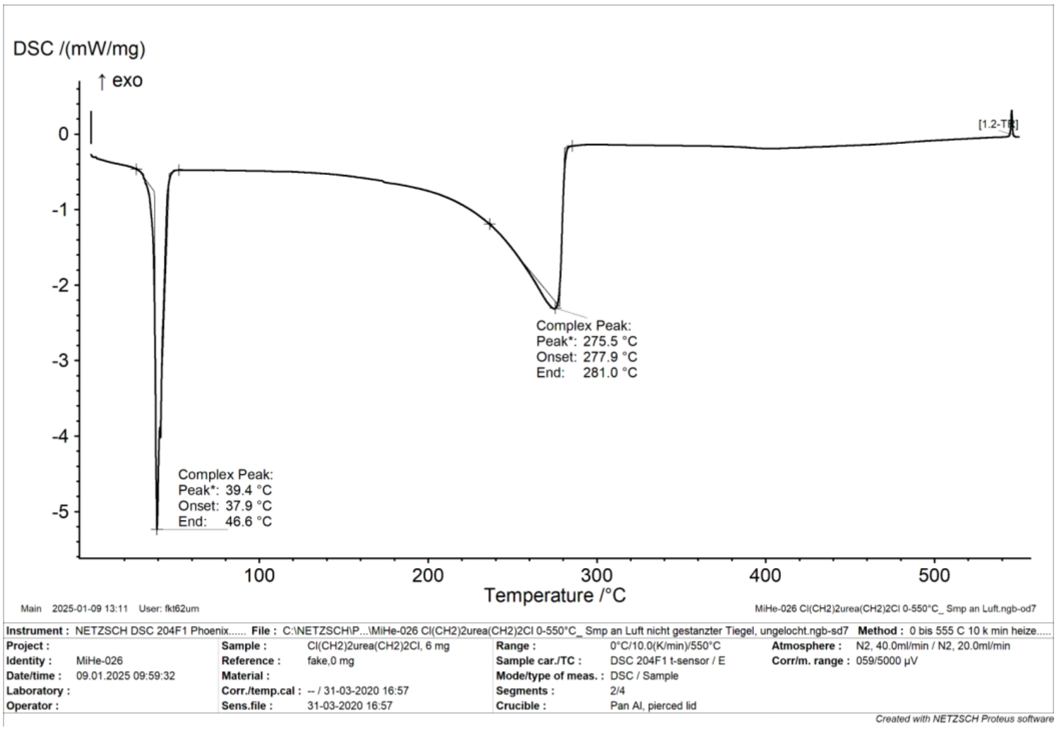

Fig. S7. DSC curve of **5**.

1,3-Divinylimidazolidin-2-one (DVI)

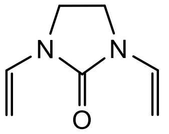

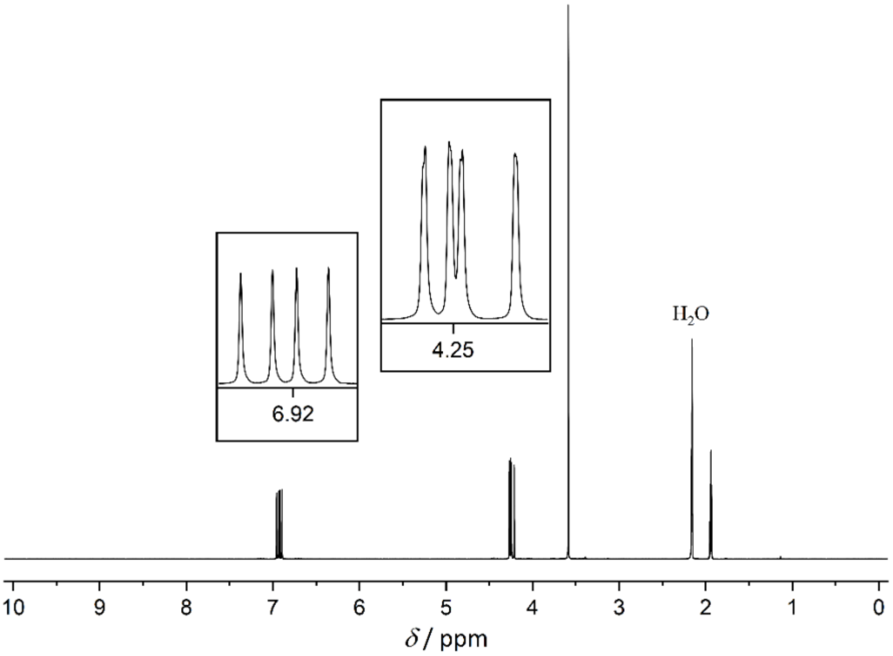

Fig. S8a. ^{1}H NMR spectrum of **DVI** in CD_3CN.

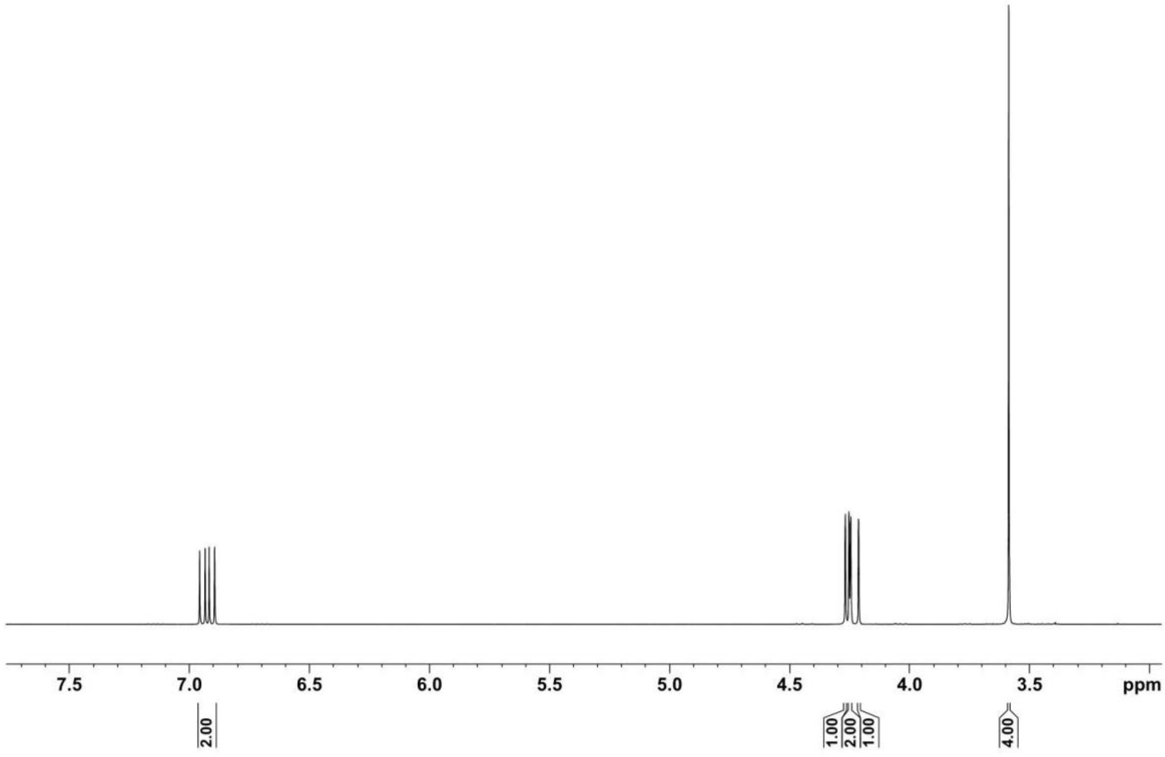

Fig. S8b. ^{1}H NMR spectrum of **DVI** in CD_3CN.

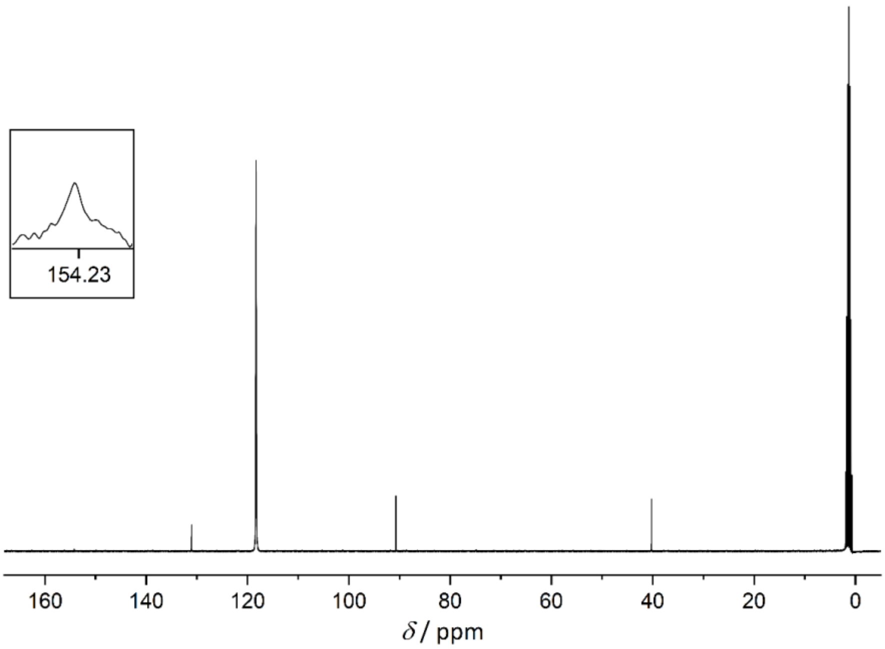

Fig. S9. $^{13}C\{^1H\}$ NMR spectrum of **DVI** in CD_3CN.

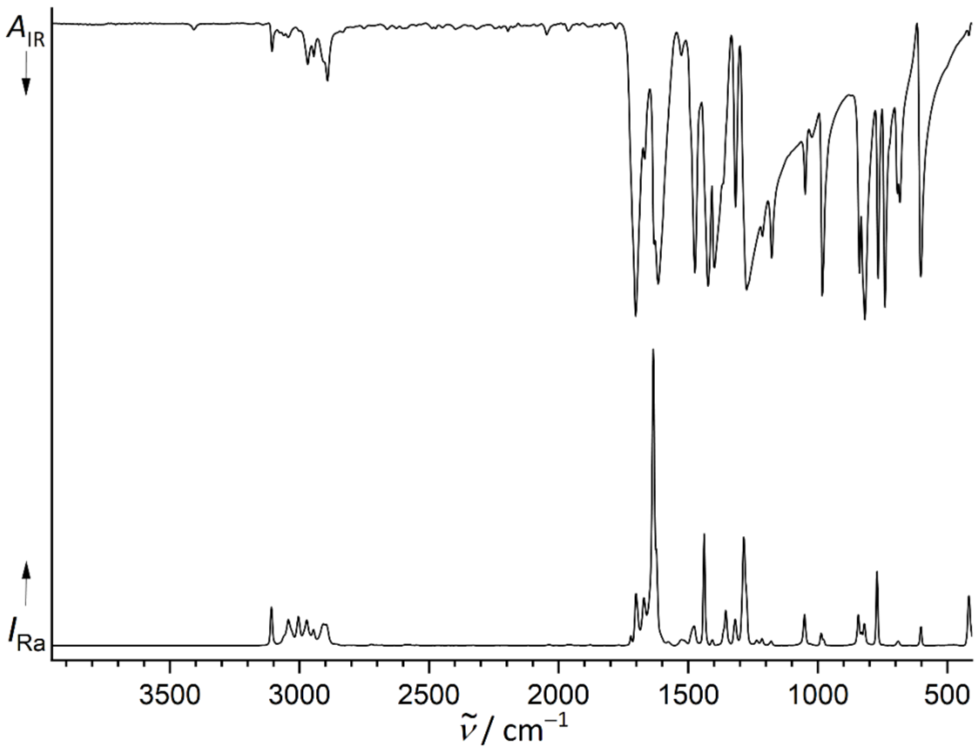

Fig. S10. IR (top) and Raman spectrum (bottom) of **DVI**.

Crystal Structure Determination

Crystal data of **5** and **DVI** were collected on a XtaLAB Synergy, Dualflex diffractometer with a hybrid pixel array detector, using $Cu_{K\alpha}$ radiation (micro-focus sealed X-ray tube, λ_{Cu} = 1.54184 Å). The structures were solved by intrinsic phasing methods (SHELXT).[1-2] Refinement is based on full-matrix least-squares calculations on F^2 (SHELXL).[2-3] All non-hydrogen atoms were refined anisotropically. Unless specified differently, for CH idealized bond lengths and angles were used. Calculations were carried out using the ShelXle graphical interface.[4] Molecular structure diagrams were drawn with the program Diamond 5.1.0.[5]

Crystallographic data have been deposited with the Cambridge Crystallographic Data Centre. Experimental details, crystal data, and CCDC numbers are collected in the following table. These data can be obtained free of charge from The Cambridge Crystallographic Data Centre via www.ccdc.cam.ac.uk/data_request/cif.

Table S1. Selected crystal data and details of the refinement of the crystal structures of 1,3-di(2-chloroethyl)imidazolidin-2-one (**5**) and 1,3-divinylimidazolidin-2-one (**DVI**).

Data	**5**	**DVI**
Empirical formula	$C_7H_{12}Cl_2N_2O$	$C_7H_{10}N_2O$
Formula weight (g·mol^{-1})	211.09	138.17
Temperature (K)	100(2)	100(2)
Radiation, *l* (Å)	Cu_{Ka}, 1.54184	Cu_{Ka}, 1.54184
Crystal system	monoclinic	monoclinic
Space group	$P2_1/n$	$P2_1/c$
Unit cell dimensions		
a (Å)	8.80990(10)	8.48580(10)
b (Å)	6.45850(10)	8.21980(10)
c (Å)	17.4459(2)	10.69410(10)
a (°)	90	90
b (°)	103.8760(10)	93.6100(10)
g (°)	0.220	0.290
Volume (Å^3)	963.68(2)	744.450(14)
Z	4	4
Calculated density (Mg·m^{-3})	1.455	1.233
Absorption coefficient (mm^{-1})	5.714	0.691
F(000)	440	296
Theta range for collection	5.205 to 77.574°	5.223 to 77.654°
Reflections collected	18985	15084
Unique reflections	2038	1568
Unique reflections with [I>2s(I)]	1924	1456
Minimum/maximum transmission	0.042/0.556	0.042/0.556
Refinement method	Full-matrix least-squares on F^2	Full-matrix least-squares on F^2
Data / parameters / restraints	2038 / 109 / 0	1568 / 91 / 0
Goodness-of-fit on F^2	1.092	1.053
Final *R* indices [*I*>2*s*(*I*)]	R_1 = 0.0471, wR_2 = 0.1288	R_1 = 0.0356, wR_2 = 0.0950
R indices (all data)	R_1 = 0.0484, wR_2 = 0.1307	R_1 = 0.0374, wR_2 = 0.0965
Maximum/minimum residual electron density (e·Å^{-3})	1.059 / –0.563	0.171 / –0.252
CCDC	2494199	2494200

References

[1] SHELXT, Program for Crystal Structure Solution, **2014**.

[2] G. M. Sheldrick, *Acta Cryst.* **2008**, *A64*, 112–122.

[3] SHELXL-97, Program for Crystal Structure Refinement, 1997.

[4] C. B. Hübschle, G. M. Sheldrick, B. Dittrich, *J. Appl. Crystallogr.* **2011**, *44*, 1281–1284.

[5] Diamond 5.0.0, K. Brandenburg, Crystal Impact GbR, 1997–2023.
